# Propyl 2-(5-iodo-3-methyl­sulfinyl-1-benzofuran-2-yl)acetate

**DOI:** 10.1107/S1600536808042359

**Published:** 2008-12-17

**Authors:** Hong Dae Choi, Pil Ja Seo, Byeng Wha Son, Uk Lee

**Affiliations:** a Department of Chemistry, Dongeui University, San 24 Kaya-dong Busanjin-gu, Busan 614-714, Republic of Korea; b Department of Chemistry, Pukyong National University, 599-1 Daeyeon 3-dong Nam-gu, Busan 608-737, Republic of Korea

## Abstract

In the title compound, C_14_H_15_IO_4_S, the O atom and the methyl group of the methyl­sulfinyl substituent lie on opposite sides of the plane of the benzofuran ring system. The crystal structure is stabilized by inter­molecular C—H⋯π inter­actions between an H atom of the propyl methyl­ene group closest to the carboxyl­ate O atom and the benzene ring of a neighbouring mol­ecule, and between an H atom of the outer propyl methyl­ene group and the furan ring of a neighbouring mol­ecule, respectively. Additionally, the crystal structure exhibits inter­molecular C—H⋯O hydrogen bonds.

## Related literature

For the synthesis and crystal structures of similar alkyl 2-(5-iodo-3-methyl­sulfinyl-1-benzofuran-2-yl)acetate derivatives. see: Choi *et al.* (2007 ▶, 2008 ▶).
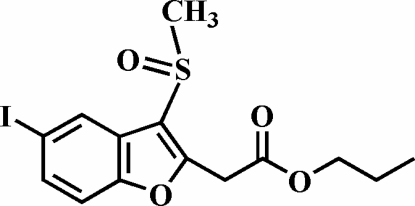


## Experimental

### 

#### Crystal data

C_14_H_15_IO_4_S
*M* *_r_* = 406.22
Triclinic, 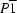

*a* = 8.5468 (4) Å
*b* = 10.0329 (5) Å
*c* = 10.3239 (5) Å
α = 72.442 (1)°
β = 81.345 (1)°
γ = 65.088 (1)°
*V* = 765.21 (6) Å^3^
*Z* = 2
Mo *K*α radiation
μ = 2.24 mm^−1^
*T* = 293 (2) K
0.30 × 0.20 × 0.10 mm

#### Data collection

Bruker SMART CCD diffractometer
Absorption correction: multi-scan (*SADABS*; Sheldrick, 1999 ▶) *T*_min_ = 0.583, *T*_max_ = 0.787
6182 measured reflections
2982 independent reflections
2778 reflections with *I* > 2σ(*I*)
*R*_int_ = 0.016

#### Refinement

*R*[*F* ^2^ > 2σ(*F* ^2^)] = 0.029
*wR*(*F* ^2^) = 0.069
*S* = 1.18
2982 reflections
183 parameters
H-atom parameters constrained
Δρ_max_ = 0.52 e Å^−3^
Δρ_min_ = −0.56 e Å^−3^

### 

Data collection: *SMART* (Bruker, 2001 ▶); cell refinement: *SAINT* (Bruker, 2001 ▶); data reduction: *SAINT*; program(s) used to solve structure: *SHELXS97* (Sheldrick, 2008 ▶); program(s) used to refine structure: *SHELXL97* (Sheldrick, 2008 ▶); molecular graphics: *ORTEP-3* (Farrugia, 1997 ▶) and *DIAMOND* (Brandenburg, 1998 ▶); software used to prepare material for publication: *SHELXL97*.

## Supplementary Material

Crystal structure: contains datablocks global, I. DOI: 10.1107/S1600536808042359/sj2566sup1.cif

Structure factors: contains datablocks I. DOI: 10.1107/S1600536808042359/sj2566Isup2.hkl

Additional supplementary materials:  crystallographic information; 3D view; checkCIF report

## Figures and Tables

**Table 1:** Hydrogen-bond geometry (Å, °)

*D*—H⋯*A*	*D*—H	H⋯*A*	*D*⋯*A*	*D*—H⋯*A*
C11—H11*B*⋯*Cg*1^i^	0.97	3.12	3.814 (4)	130
C12—H12*B*⋯*Cg*2^ii^	0.97	2.99	3.929 (3)	162
C3—H3⋯O4^iii^	0.93	2.60	3.468 (4)	157
C9—H9*B*⋯O4^iv^	0.97	2.40	3.356 (4)	167

Symmetry codes: (i) 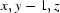; (ii) 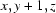; (iii) 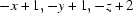; (iv) 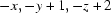. *Cg*1 and *Cg*2 are the centroids of the C2–C7 benzene ring and the C1/C2/C7/O1/C8 furan ring, respectively.

**Fig. 1.**
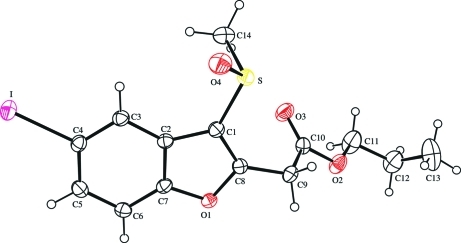
The molecular structure of the title compound, showing displacement ellipsoids drawn at the 30% probability level.

**Fig. 2.**
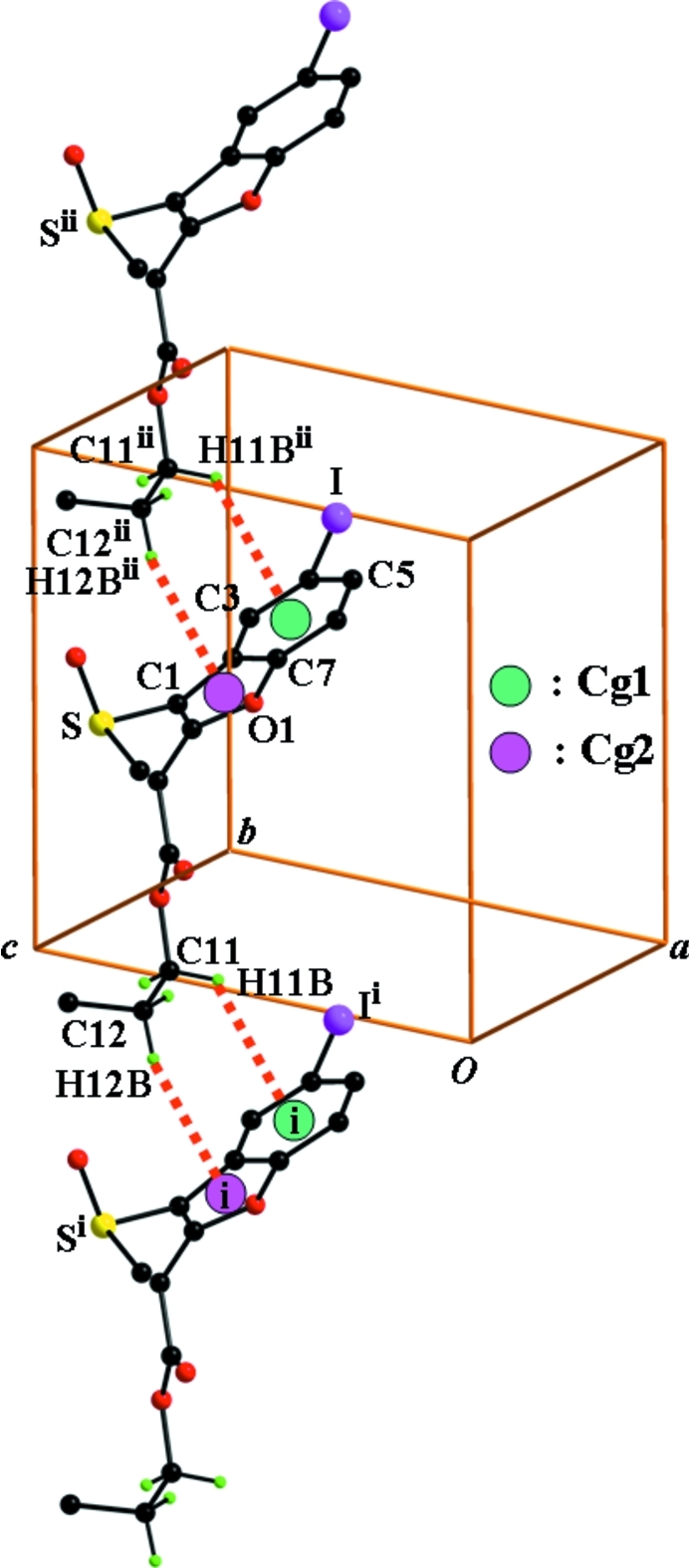
C—H···π interactions (dotted lines) in the title compound. Cg denotes the ring centroid.[Symmetry code: (i) x, y-1, z; (ii) x, y+1, z.]

## supplementary crystallographic information

### Comment

This work is related to our previous communications on the synthesis and structure of alkyl 2-(5-iodo-3-methylsulfinyl-1-benzofuran-2-yl)acetate analogues, *viz*. ethyl 2-(5-iodo-3-methylsulfinyl-1-benzofuran-2-yl)acetate (Choi *et al.*, 2007) and isopropyl 2-(5-iodo-3-methylsulfinyl-1-benzofuran-2-yl)acetate (Choi *et al.*, 2008). Here we report the crystal structure of the title compound, propyl 2-(5-iodo-3-methylsulfinyl-1-benzofuran-2-yl)acetate (Fig. 1).

The benzofuran unit is essentially planar, with a mean deviation of 0.012 (2) Å from the least-squares plane defined by the nine constituent atoms. The molecular packing (Fig. 2) is stabilized by intermolecular C—H···π interactions within each stack of molecules; one between the hydrogen of 11–methylene group and the benzene ring of the benzofuran unit, with a C11—H11B···Cg1^i^ separation of 3.12 Å, and a second between the hydrogen of 12-methylene group and the furan ring of the benzofuran unit, with a C12—H12B···Cg2^i^ with a separation of 2.99 Å (Table 1 and Fig. 2; *Cg*1 and *Cg*2 are the centroids of the C2–C7 benzene ring and the C1/C2/C7/O1/C8 furan ring, respectively, symmetry code as in Fig. 2). In addition, intermolecular C—H···O hydrogen bonds in the structure are observed (Table 1).

### Experimental

77% 3-chloroperoxybenzoic acid (173 mg, 0.77 mmol) was added in small portions to a stirred solution of propyl 2-(5-iodo-3-methylsulfanyl-1-benzofuran-2-yl)acetate (273 mg, 0.7 mmol) in dichloromethane (30 ml) at 273 K. After stirring for 3 h at room temperature, the mixture was washed with saturated sodium bicarbonate solution and the organic layer was separated, dried over magnesium sulfate, filtered and concentrated in vacuum. The residue was purified by column chromatography (hexane-ethyl acetate, 1:2 v/v) to afford the title compound as a colorless solid [yield 80%, m.p. 412-413 K; *R*_f_ = 0.58 (hexane-ethyl acetate, 1;2 v/v)]. Single crystals suitable for X-ray diffraction were prepared by evaporation of a solution of the title compound in acetone at room temperature. Spectroscopic analysis: ^1^H NMR (CDCl_3_, 400 MHz) δ 0.93 (t, J = 7.36 Hz, 3H), 1.64-1.72 (m, 2H), 3.07 (s, 3H), 4.02 (s, 2H), 4.11 (t, J = 6.60 Hz, 2H), 7.29 (d, J = 8.76 Hz, 1H), 7.66 (dd, J = 8.80 Hz and J = 1.84 Hz, 1H), 8.28 (d, J = 1.48 Hz, 1H); EI-MS 406 [M^+^].

### Refinement

All H atoms were positioned geometrically and refined using a riding model, with C—H = 0.93 Å for the aryl, 0.97 Å for the methylene, and 0.96 Å for the methyl H atoms. *U*iso(H) = 1.2*U*eq(C) for the aryl and methylene H atoms, and 1.5*U*eq(C) for methyl H atoms.

### Crystal data

**Table:**

C_14_H_15_IO_4_S	*Z* = 2
*M**_r_* = 406.22	*F*(000) = 400
Triclinic, *P*1	*D*_x_ = 1.763 Mg m^−^^3^
Hall symbol: -P 1	Mo *K*α radiation, λ = 0.71073 Å
*a* = 8.5468 (4) Å	Cell parameters from 4625 reflections
*b* = 10.0329 (5) Å	θ = 2.3–28.3°
*c* = 10.3239 (5) Å	µ = 2.24 mm^−^^1^
α = 72.442 (1)°	*T* = 293 K
β = 81.345 (1)°	Plate, colorless
γ = 65.088 (1)°	0.30 × 0.20 × 0.10 mm
*V* = 765.21 (6) Å^3^	

### Data collection

**Table:**

Bruker SMART CCD diffractometer	2982 independent reflections
Radiation source: fine-focus sealed tube	2778 reflections with *I* > 2σ(*I*)
graphite	*R*_int_ = 0.016
Detector resolution: 10.0 pixels mm^-1^	θ_max_ = 26.0°, θ_min_ = 2.3°
φ and ω scans	*h* = −10→10
Absorption correction: multi-scan (*SADABS*; Sheldrick, 1999)	*k* = −12→12
*T*_min_ = 0.583, *T*_max_ = 0.787	*l* = −12→12
6182 measured reflections	

### Refinement

**Table:**

Refinement on *F*^2^	Primary atom site location: structure-invariant direct methods
Least-squares matrix: full	Secondary atom site location: difference Fourier map
*R*[*F*^2^ > 2σ(*F*^2^)] = 0.029	Hydrogen site location: difference Fourier map
*wR*(*F*^2^) = 0.069	H-atom parameters constrained
*S* = 1.18	*w* = 1/[σ^2^(*F*_o_^2^) + (0.0293*P*)^2^ + 0.5734*P*] where *P* = (*F*_o_^2^ + 2*F*_c_^2^)/3
2982 reflections	(Δ/σ)_max_ = 0.001
183 parameters	Δρ_max_ = 0.52 e Å^−^^3^
0 restraints	Δρ_min_ = −0.56 e Å^−^^3^

### Special details

**Table:**

Geometry. All esds (except the esd in the dihedral angle between two l.s. planes) are estimated using the full covariance matrix. The cell esds are taken into account individually in the estimation of esds in distances, angles and torsion angles; correlations between esds in cell parameters are only used when they are defined by crystal symmetry. An approximate (isotropic) treatment of cell esds is used for estimating esds involving l.s. planes.
Refinement. Refinement of F^2^ against ALL reflections. The weighted R-factor wR and goodness of fit S are based on F^2^, conventional R-factors R are based on F, with F set to zero for negative F^2^. The threshold expression of F^2^ > 2sigma(F^2^) is used only for calculating R-factors(gt) etc. and is not relevant to the choice of reflections for refinement. R-factors based on F^2^ are statistically about twice as large as those based on F, and R- factors based on ALL data will be even larger.

### Fractional atomic coordinates and isotropic or equivalent isotropic displacement parameters (Å^2^)

**Table:**

	*x*	*y*	*z*	*U*_iso_*/*U*_eq_	
I	0.69366 (3)	0.79269 (3)	0.61486 (2)	0.04832 (10)	
S	0.26183 (10)	0.40380 (10)	0.96397 (8)	0.03932 (18)	
O1	0.1660 (3)	0.5368 (2)	0.5730 (2)	0.0350 (4)	
O2	0.0455 (3)	0.1416 (3)	0.7355 (3)	0.0530 (6)	
O3	0.2911 (3)	0.1379 (3)	0.7896 (3)	0.0567 (7)	
O4	0.2582 (3)	0.5257 (3)	1.0206 (2)	0.0529 (6)	
C1	0.2539 (4)	0.4770 (3)	0.7857 (3)	0.0337 (6)	
C2	0.3414 (4)	0.5677 (3)	0.6976 (3)	0.0303 (6)	
C3	0.4622 (4)	0.6215 (3)	0.7127 (3)	0.0335 (6)	
H3	0.5072	0.5992	0.7969	0.040*	
C4	0.5121 (4)	0.7091 (3)	0.5973 (3)	0.0340 (6)	
C5	0.4466 (4)	0.7459 (3)	0.4690 (3)	0.0387 (7)	
H5	0.4825	0.8069	0.3943	0.046*	
C6	0.3287 (4)	0.6913 (3)	0.4533 (3)	0.0368 (7)	
H6	0.2844	0.7134	0.3689	0.044*	
C7	0.2796 (4)	0.6029 (3)	0.5682 (3)	0.0322 (6)	
C8	0.1529 (4)	0.4619 (3)	0.7075 (3)	0.0330 (6)	
C9	0.0405 (4)	0.3755 (3)	0.7359 (3)	0.0367 (7)	
H9A	−0.0360	0.4135	0.6606	0.044*	
H9B	−0.0303	0.3929	0.8168	0.044*	
C10	0.1438 (4)	0.2060 (4)	0.7564 (3)	0.0396 (7)	
C11	0.1272 (5)	−0.0221 (4)	0.7496 (6)	0.0702 (13)	
H11A	0.1879	−0.0747	0.8343	0.084*	
H11B	0.2094	−0.0435	0.6752	0.084*	
C12	−0.0126 (6)	−0.0740 (5)	0.7479 (5)	0.0683 (12)	
H12A	−0.0746	−0.0170	0.6641	0.082*	
H12B	0.0396	−0.1808	0.7486	0.082*	
C13	−0.1370 (9)	−0.0548 (7)	0.8641 (6)	0.102 (2)	
H13A	−0.0787	−0.1189	0.9471	0.154*	
H13B	−0.2278	−0.0829	0.8536	0.154*	
H13C	−0.1850	0.0497	0.8670	0.154*	
C14	0.4798 (5)	0.2651 (4)	0.9739 (4)	0.0539 (9)	
H14A	0.5572	0.3159	0.9444	0.081*	
H14B	0.4958	0.2005	0.9166	0.081*	
H14C	0.5031	0.2045	1.0661	0.081*	

### Atomic displacement parameters (Å^2^)

**Table:**

	*U*^11^	*U*^22^	*U*^33^	*U*^12^	*U*^13^	*U*^23^
I	0.04655 (14)	0.04768 (14)	0.05934 (16)	−0.02881 (11)	−0.00540 (10)	−0.00948 (10)
S	0.0427 (4)	0.0487 (4)	0.0301 (4)	−0.0235 (4)	−0.0015 (3)	−0.0075 (3)
O1	0.0358 (11)	0.0404 (11)	0.0329 (11)	−0.0184 (9)	−0.0071 (8)	−0.0077 (9)
O2	0.0399 (12)	0.0362 (12)	0.0874 (19)	−0.0165 (10)	−0.0083 (12)	−0.0177 (12)
O3	0.0393 (13)	0.0466 (14)	0.0806 (19)	−0.0156 (11)	−0.0146 (12)	−0.0079 (13)
O4	0.0619 (16)	0.0638 (16)	0.0409 (13)	−0.0264 (13)	−0.0003 (11)	−0.0234 (12)
C1	0.0349 (15)	0.0350 (15)	0.0336 (15)	−0.0152 (13)	−0.0026 (12)	−0.0098 (12)
C2	0.0314 (14)	0.0304 (14)	0.0297 (14)	−0.0112 (12)	−0.0026 (11)	−0.0099 (11)
C3	0.0360 (15)	0.0335 (15)	0.0339 (16)	−0.0142 (12)	−0.0052 (12)	−0.0104 (12)
C4	0.0316 (15)	0.0311 (14)	0.0419 (16)	−0.0131 (12)	−0.0016 (12)	−0.0119 (13)
C5	0.0400 (17)	0.0335 (15)	0.0377 (16)	−0.0138 (13)	0.0009 (13)	−0.0049 (13)
C6	0.0392 (16)	0.0395 (16)	0.0301 (15)	−0.0150 (13)	−0.0048 (12)	−0.0062 (13)
C7	0.0299 (14)	0.0311 (14)	0.0378 (15)	−0.0111 (12)	−0.0053 (12)	−0.0118 (12)
C8	0.0339 (15)	0.0320 (15)	0.0335 (15)	−0.0142 (12)	−0.0027 (12)	−0.0070 (12)
C9	0.0343 (15)	0.0412 (16)	0.0410 (17)	−0.0188 (13)	−0.0023 (13)	−0.0134 (13)
C10	0.0397 (17)	0.0422 (17)	0.0403 (17)	−0.0220 (14)	0.0007 (13)	−0.0082 (14)
C11	0.051 (2)	0.038 (2)	0.122 (4)	−0.0153 (17)	−0.001 (2)	−0.027 (2)
C12	0.063 (3)	0.043 (2)	0.105 (4)	−0.0231 (19)	−0.004 (2)	−0.026 (2)
C13	0.124 (5)	0.100 (4)	0.111 (5)	−0.078 (4)	0.044 (4)	−0.040 (4)
C14	0.051 (2)	0.055 (2)	0.049 (2)	−0.0147 (17)	−0.0128 (16)	−0.0084 (17)

### Geometric parameters (Å, °)

**Table:**

I—C4	2.102 (3)	C6—C7	1.379 (4)
S—O4	1.494 (3)	C6—H6	0.9300
S—C1	1.764 (3)	C8—C9	1.491 (4)
S—C14	1.790 (4)	C9—C10	1.513 (4)
O1—C7	1.375 (3)	C9—H9A	0.9700
O1—C8	1.378 (3)	C9—H9B	0.9700
O2—C10	1.329 (4)	C11—C12	1.495 (6)
O2—C11	1.458 (4)	C11—H11A	0.9700
O3—C10	1.197 (4)	C11—H11B	0.9700
C1—C8	1.347 (4)	C12—C13	1.480 (7)
C1—C2	1.443 (4)	C12—H12A	0.9700
C2—C3	1.397 (4)	C12—H12B	0.9700
C2—C7	1.399 (4)	C13—H13A	0.9600
C3—C4	1.380 (4)	C13—H13B	0.9600
C3—H3	0.9300	C13—H13C	0.9600
C4—C5	1.400 (4)	C14—H14A	0.9600
C5—C6	1.381 (4)	C14—H14B	0.9600
C5—H5	0.9300	C14—H14C	0.9600
			
O4—S—C1	106.92 (14)	C10—C9—H9A	109.1
O4—S—C14	106.66 (17)	C8—C9—H9B	109.1
C1—S—C14	98.06 (16)	C10—C9—H9B	109.1
C7—O1—C8	106.0 (2)	H9A—C9—H9B	107.9
C10—O2—C11	116.8 (3)	O3—C10—O2	124.3 (3)
C8—C1—C2	107.3 (3)	O3—C10—C9	125.7 (3)
C8—C1—S	123.9 (2)	O2—C10—C9	110.0 (3)
C2—C1—S	128.7 (2)	O2—C11—C12	107.4 (3)
C3—C2—C7	119.1 (3)	O2—C11—H11A	110.2
C3—C2—C1	136.3 (3)	C12—C11—H11A	110.2
C7—C2—C1	104.6 (2)	O2—C11—H11B	110.2
C4—C3—C2	117.2 (3)	C12—C11—H11B	110.2
C4—C3—H3	121.4	H11A—C11—H11B	108.5
C2—C3—H3	121.4	C13—C12—C11	113.5 (4)
C3—C4—C5	123.1 (3)	C13—C12—H12A	108.9
C3—C4—I	118.6 (2)	C11—C12—H12A	108.9
C5—C4—I	118.3 (2)	C13—C12—H12B	108.9
C6—C5—C4	119.9 (3)	C11—C12—H12B	108.9
C6—C5—H5	120.1	H12A—C12—H12B	107.7
C4—C5—H5	120.1	C12—C13—H13A	109.5
C7—C6—C5	117.2 (3)	C12—C13—H13B	109.5
C7—C6—H6	121.4	H13A—C13—H13B	109.5
C5—C6—H6	121.4	C12—C13—H13C	109.5
O1—C7—C6	125.8 (3)	H13A—C13—H13C	109.5
O1—C7—C2	110.7 (3)	H13B—C13—H13C	109.5
C6—C7—C2	123.6 (3)	S—C14—H14A	109.5
C1—C8—O1	111.4 (3)	S—C14—H14B	109.5
C1—C8—C9	133.3 (3)	H14A—C14—H14B	109.5
O1—C8—C9	115.2 (2)	S—C14—H14C	109.5
C8—C9—C10	112.3 (2)	H14A—C14—H14C	109.5
C8—C9—H9A	109.1	H14B—C14—H14C	109.5
			
O4—S—C1—C8	−135.7 (3)	C3—C2—C7—O1	178.3 (2)
C14—S—C1—C8	114.1 (3)	C1—C2—C7—O1	−1.5 (3)
O4—S—C1—C2	40.8 (3)	C3—C2—C7—C6	−1.4 (4)
C14—S—C1—C2	−69.4 (3)	C1—C2—C7—C6	178.8 (3)
C8—C1—C2—C3	−178.7 (3)	C2—C1—C8—O1	−0.3 (3)
S—C1—C2—C3	4.4 (5)	S—C1—C8—O1	176.8 (2)
C8—C1—C2—C7	1.0 (3)	C2—C1—C8—C9	175.8 (3)
S—C1—C2—C7	−175.9 (2)	S—C1—C8—C9	−7.1 (5)
C7—C2—C3—C4	0.8 (4)	C7—O1—C8—C1	−0.6 (3)
C1—C2—C3—C4	−179.4 (3)	C7—O1—C8—C9	−177.5 (2)
C2—C3—C4—C5	0.4 (4)	C1—C8—C9—C10	−74.2 (4)
C2—C3—C4—I	−180.0 (2)	O1—C8—C9—C10	101.8 (3)
C3—C4—C5—C6	−1.2 (5)	C11—O2—C10—O3	−2.4 (5)
I—C4—C5—C6	179.2 (2)	C11—O2—C10—C9	179.2 (3)
C4—C5—C6—C7	0.7 (4)	C8—C9—C10—O3	22.6 (5)
C8—O1—C7—C6	−179.0 (3)	C8—C9—C10—O2	−159.0 (3)
C8—O1—C7—C2	1.3 (3)	C10—O2—C11—C12	169.5 (4)
C5—C6—C7—O1	−179.1 (3)	O2—C11—C12—C13	−63.9 (6)
C5—C6—C7—C2	0.6 (4)		

### Hydrogen-bond geometry (Å, °)

**Table:**

*D*—H···*A*	*D*—H	H···*A*	*D*···*A*	*D*—H···*A*
C11—H11B···Cg1^i^	0.97	3.12	3.814 (4)	130
C12—H12B···Cg2^ii^	0.97	2.99	3.929 (3)	162
C3—H3···O4^iii^	0.93	2.60	3.468 (4)	157
C9—H9B···O4^iv^	0.97	2.40	3.356 (4)	167

Symmetry codes: (i) *x*, *y*−1, *z*; (ii) *x*, *y*+1, *z*; (iii) −*x*+1, −*y*+1, −*z*+2; (iv) −*x*, −*y*+1, −*z*+2.
